# Supplementary material for: Replication competent HIV-guided CRISPR screen identifies antiviral factors including targets of the accessory protein Nef
Source: Nat Commun. 2024 May 7;15:3813. doi: 10.1038/s41467-024-48228-x (PMC11076291; doi:10.1038/s41467-024-48228-x)
Supplement: Supplementary file 1 — Supplementary Information [file 41467_2024_48228_MOESM1_ESM.pdf]

Supplementary Information

Replication competent HIV-guided CRISPR screen identifies antiviral factors including targets of the accessory protein Nef

Caterina Prelli Bozzo[1#], Alexandre Laliberté[1#], Aurora De Luna[1], Chiara Pastorio[1], Kerstin Regensburger[1], Stefan Krebs[2], Alexander Graf[2], Helmut Blum[2], Meta Volcic[1], Konstantin M.J. Sparrer[1*] & Frank Kirchhoff[1*]

[1] Institute of Molecular Virology

Ulm University Medical Center

89081 Ulm, Germany

[2] Laboratory for Functional Genome Analysis

Gene Center, LMU Munich,

81377 Munich, Germany

[#] contributed equally

* Correspondence: Konstantin.Sparrer@uni-ulm.de and Frank.Kirchhoff@uni-ulm.de

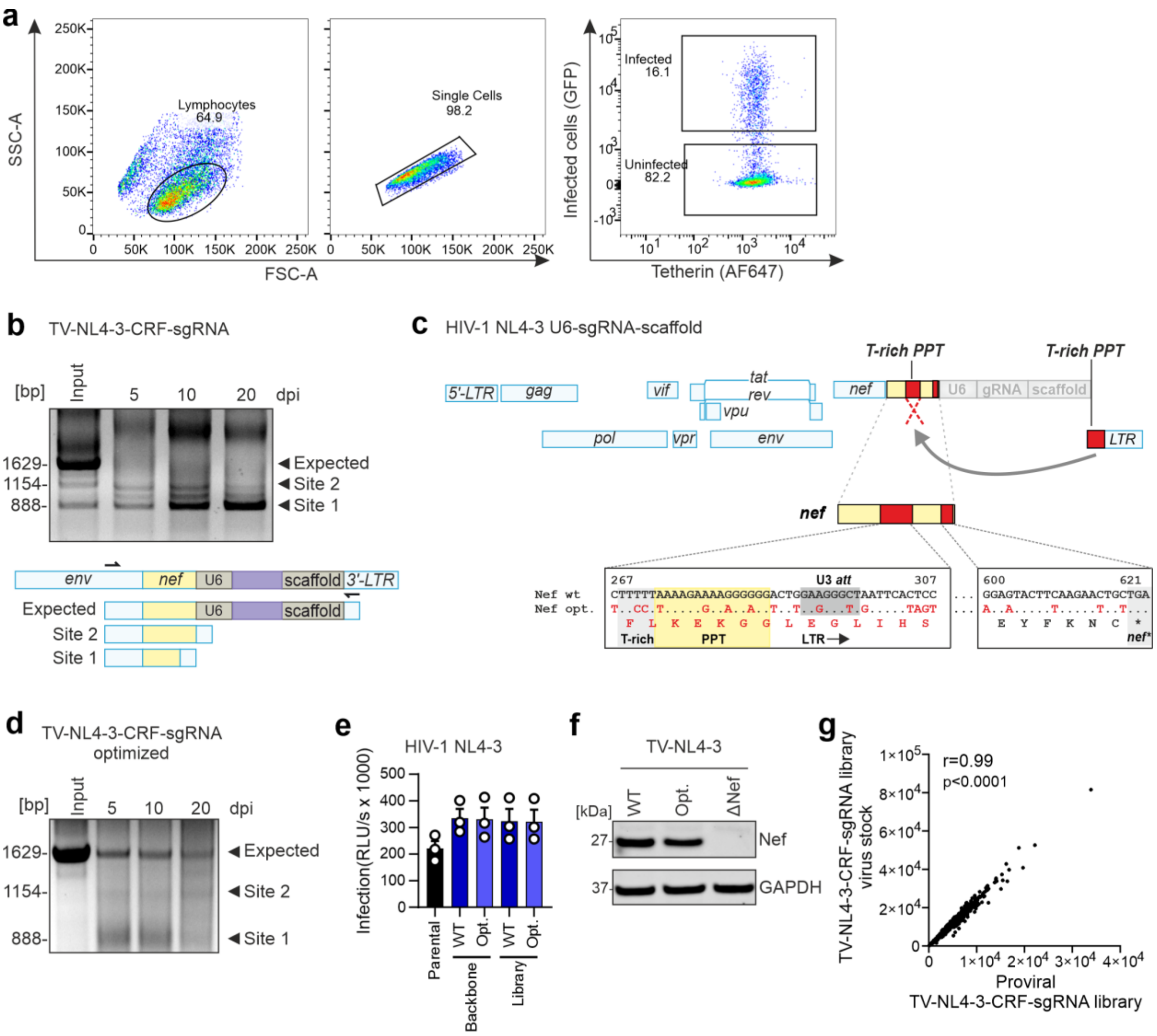

Supplementary Figure 1. Optimization of HIV-1 NL4-3 sgRNA construct. a, Representative gating strategy related to Fig. 1c. **b,** PCR analysis of the input HIV-1 sgRNA stocks and viral variants obtained at the indicated days of passaging. The upper panel shows primary PCR data and the lower panel the position of the primer binding sites and fragments obtained. **c,** Schematic structure of the HIV-1 genome and modifications at its 3´end to insert the U6-sgRNA-scaffold expression cassette. Duplicated T-rich regions, poly-purine tract (PPT) and LTR sequences are highlighted in red. The arrow indicates the major recombination event. Mutations introduced to minimize recombination and the predicted Nef amino acid sequence are indicated. Numbers refer to nucleotide positions in the NL4-3 *nef* gene. **d,** PCR analysis was performed as in panel (a) but the optimized HIV-1 NL4-3 sgRNA construct containing the changes shown in (c) were used for passaging. **e,** HEK293T cells were transfected with the parental HIV-1 NL4-3 construct (black), the original (darker blue) or optimized derivatives (lighter blue) containing the U6-sgRNA-scaffold cassette or the sgRNA library targeting 511 potential antiviral factors. Infectious virus yield was measured using the TZM-bl reporter cell infectivity assay. Bars represent the mean of three independent experiments ±SEM. **f,** Representative Western blot of Nef and GAPDH expression levels in HEK293T cells transfected with the indicated HIV-1 NL4-3 constructs. **g,** Read counts in the virus stock correlated closely with read counts in the proviral DNA of the HIV-1 NL4-3 libraries.

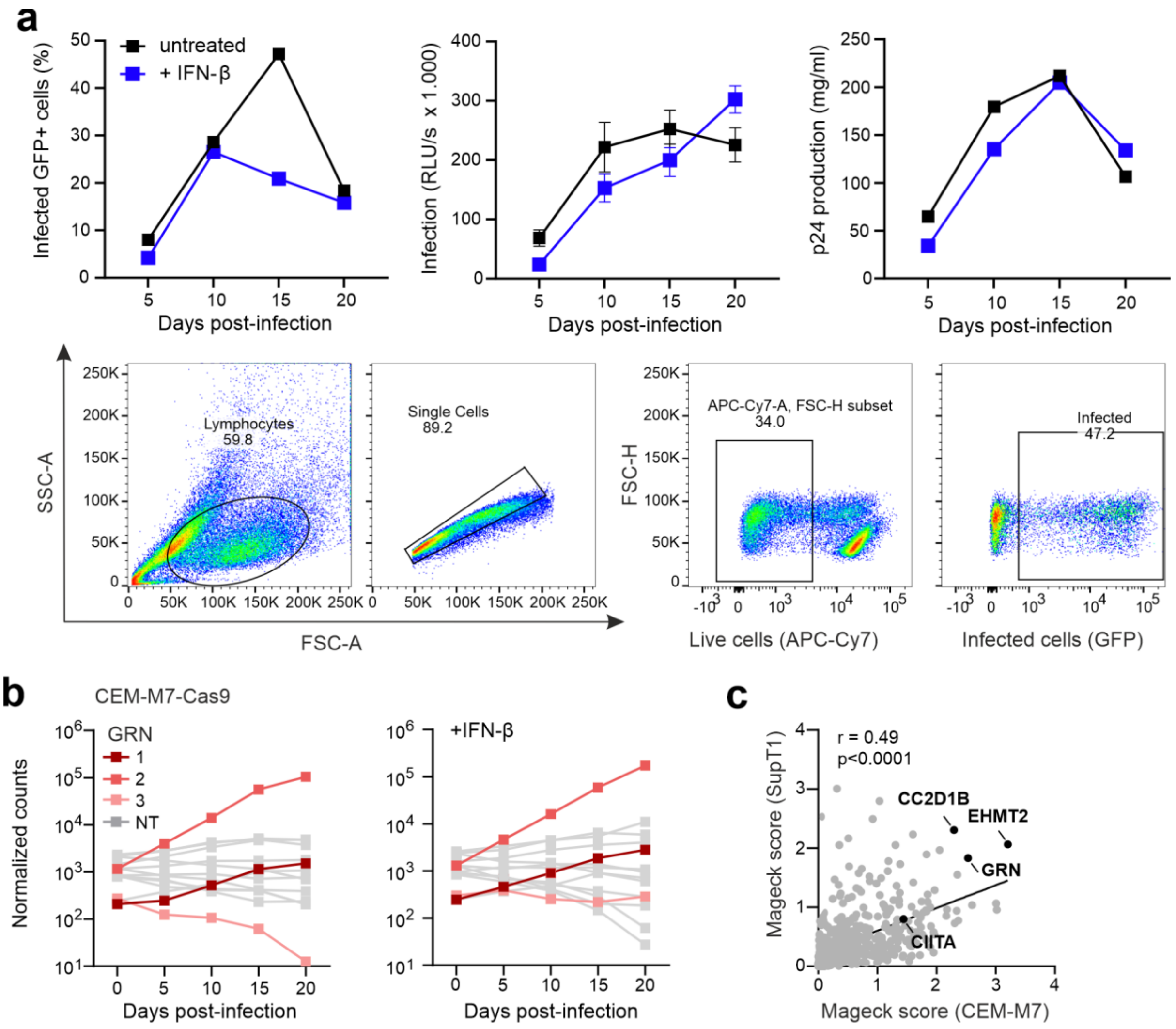

Supplementary Figure 2. Spread and enrichment of HIV-1 CRF-sgRNA constructs in cell culture. a, Infected cells, infectious virus yields, and p24 antigen production in CEM-M7 Cas9 cells infected with the HIV-1 TV-NL4-3-CRF-sgRNA library in absence (black) and presence (blue) of IFN-β. Cells were infected and the virus passaged as in Fig. 1a. Every five days, the cell cultures were analyzed for the proportion of productively infected (GFP+) cells by flow cytometry (left), infectious virus in the supernatants by TZM-bl infection assay (middle), and p24 antigen by ELISA (right). (Lower panel) Representative gating strategy. **b,** Normalized counts of the indicated HIV-1 sgRNA constructs during passage in CEM-M7 Cas9 cells (different sgRNAs are indicated in shades of red). **c,** Correlation between MAGeCK score obtained in independent experiments in CEM-M7 vs SupT1 Cas9 cells. Pearson’s Correlation, r and p values indicated.

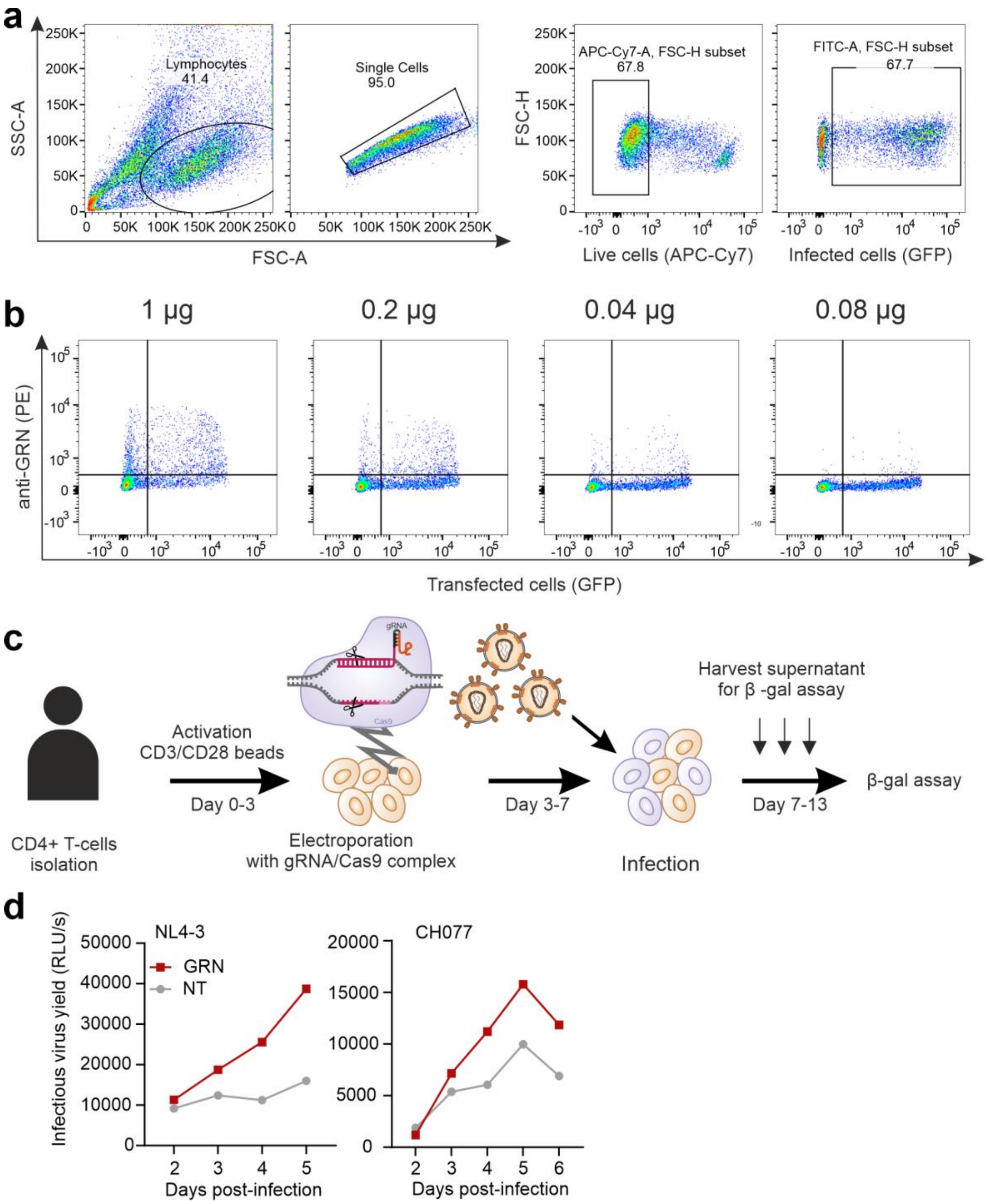

Supplementary Figure 3. Effect of GRN KO on HIV-1 replication in primary human cells. **a,** Representative gating strategy of Fig. 3b. **b,** Representative gating strategy of Fig. 3e. **c,** Schematic representation of the experimental workflow to KO target genes in primary CD4$^+$ T cells and infect them with WT HIV-1. **d,** Replication kinetics of the indicated WT HIV-1 strains in primary CD4$^+$ T cells electroporated with GRN (red) or NT (grey) sgRNA from one representative donor. Each point represents the average of the infectious virus yield measured in triplicates.

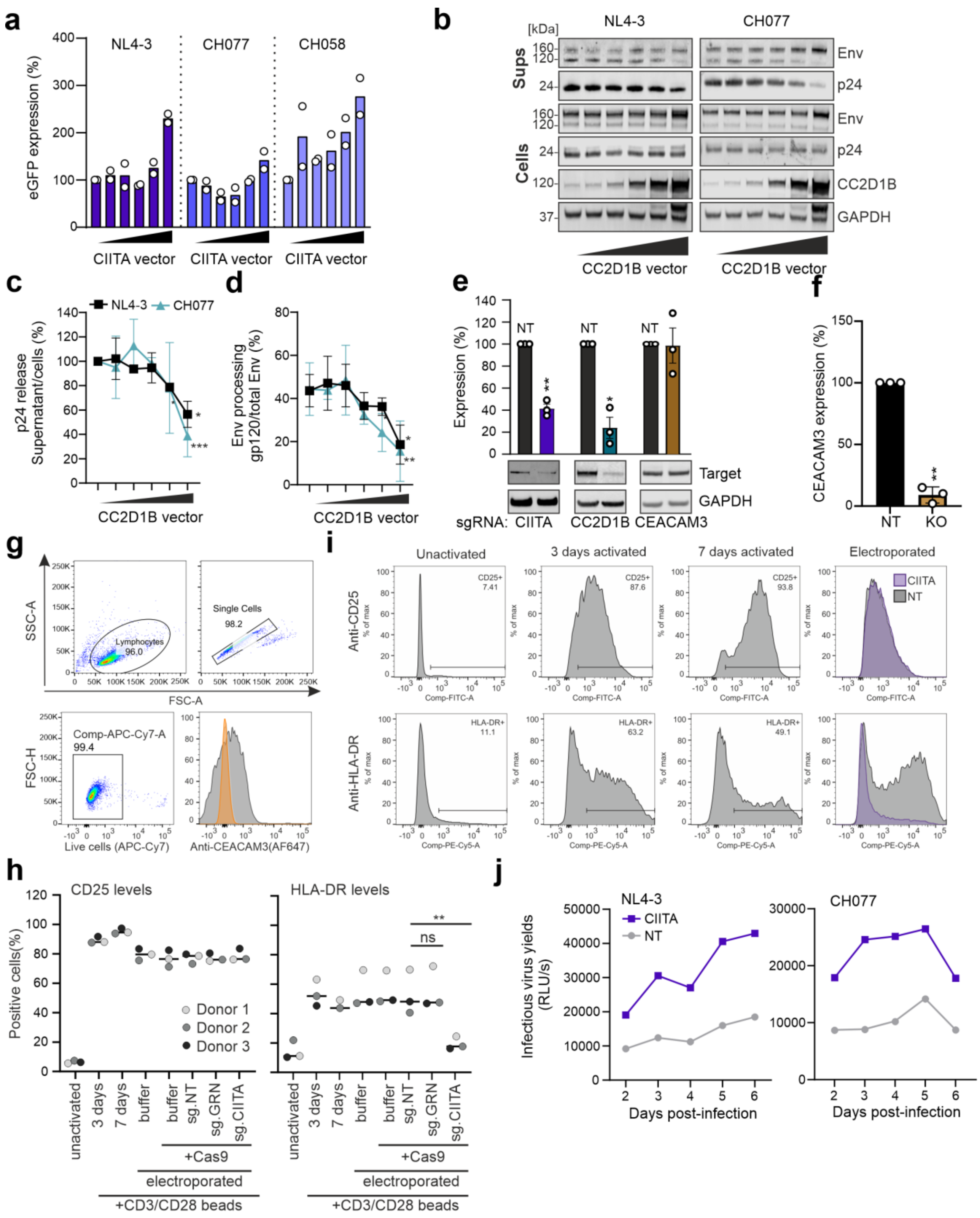

Supplementary Figure 4. Impact of CIITA and CC2D1B on HIV-1 transcription and release. a, HEK293T cells were cotransfected with increasing amounts of CIITA expression plasmid and either NL4-3_eGFP (dark purple), CH077_eGFP (purple) or CH058_eGFP (light purple) proviral constructs. LTR-driven eGFP expression was measured by flow cytometry after 48h and bars represent the mean fluorescence intensities (MFI) of eGFP in the eGFP+/AF647+ population relative to vector control (100%). Bars represent the mean of two independent experiments. **b,** Representative Western blot showing Env, p24 and CC2D1B in virus containing supernatants or cell lysates of HEK293T cells cotransfected with increasing amounts of CC2D1B and the indicated proviral constructs. **c, d,** Quantification of p24 release

(c) and Env processing (d) from three Western blots (example shown in panel b). NL4-3 (black), CH077 (light blue). Dot represent means of three independent experiments ±SD, **e,** Representative Western blot and quantification of CIITA (purple) and CC2D1B (petrol) and CEACAM3 (brown) expression upon KO in primary CD4$^+$ T cells. Bars represent the mean of three independent donors ±SEM. **f,** CEACAM3 KO efficiency measured by flow cytometry in primary CD4$^+$ T cells. Bars represent the mean of three independent donors ±SEM (NT, black and CEACAM3 KO, brown). **g,** Representative gating strategy of the data in (f). **h,** Levels of CD25 and HLA-DR determined by flow cytometry in primary CD4$^+$ T cells either left inactivated or activated with CD3/CD28 beads and electroporated or not electroporated as indicated. Dots represent three independent donors (indicated in different shades of grey) while lines represent their median. **i,** Representative gating strategy of the data in (h). **j,** Primary data of CD4$^+$ T cells electroporated with sgRNA targeting CIITA or the NT control and infected with the indicated WT HIV-1 strains. Dots represent the mean of triplicate measurements. c, d, e, f, unpaired T-test Welch´s correction, two tailed. h, Ratio paired T-test *p<0.05, ** p<0.001, ***p<0.0001.

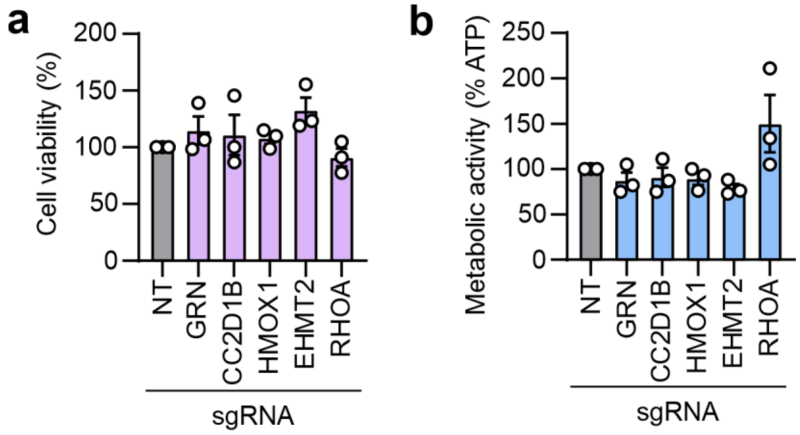

Supplementary Figure 5. Impact of sgRNA/Cas9 complex electroporation on viability of primary CD4$^+$ T cells. a, Relative cell viability, measured by MTT assay of primary CD4$^+$ T cells upon KO of GRN, CC2D1B, HMOX1, EHMT2 or RHOA. Bars represent the mean of three donors ±SEM, normalized to the cells electroporated with the NT control (grey). **b,** Relative metabolic activity, measured by CellTiter Glo assay of the cells described in panel (a). Bars represent the mean of three donors ±SEM, normalized to the cells electroporated with the NT control (grey). Unpaired T-test Welch´s correction, two tailed. no significance.

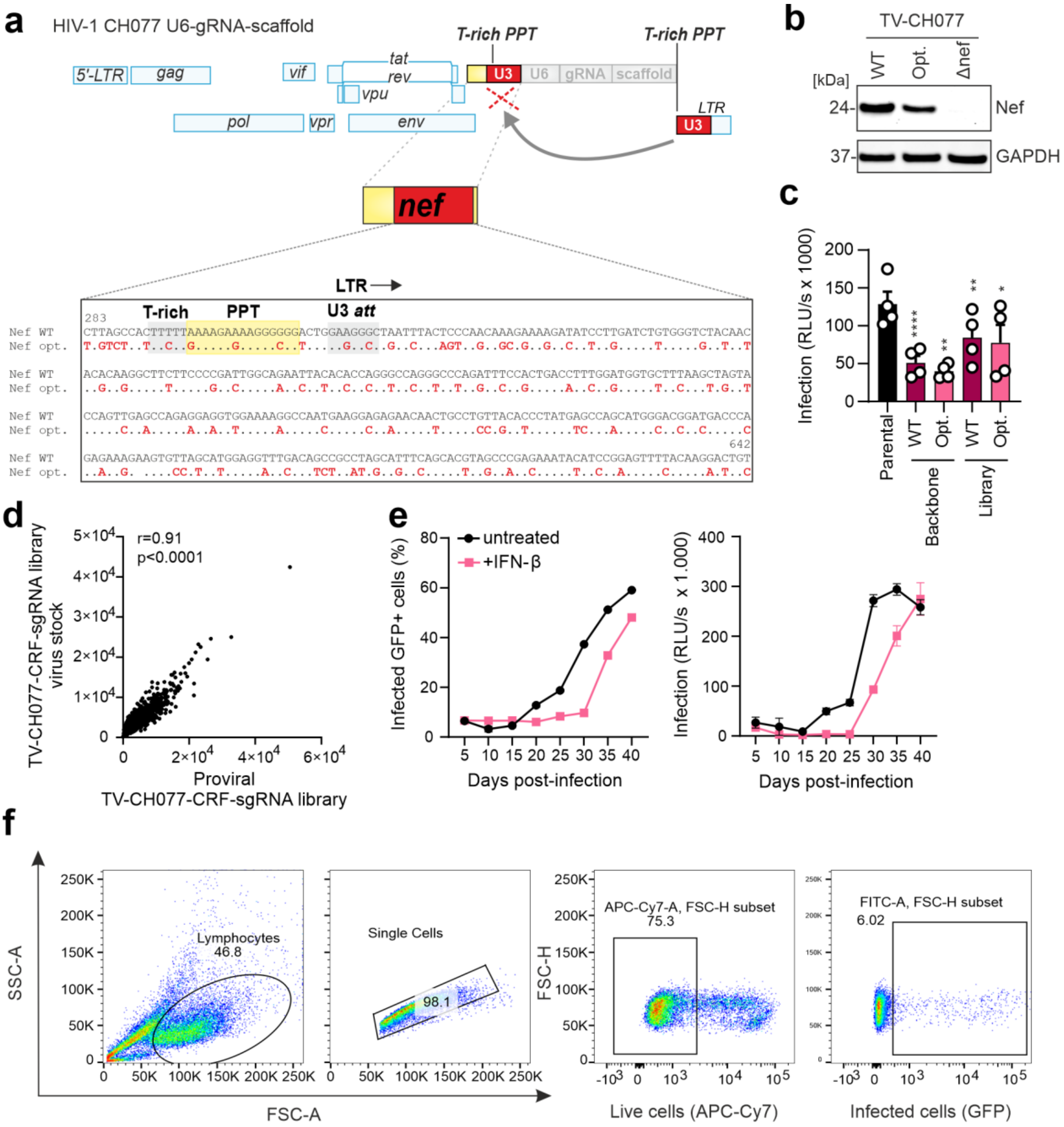

Supplementary Figure 6. Optimization of HIV-1 CH077 sgRNA constructs. a, Schematic presentation of modifications at the 3´end of the CH077 provirus to insert the U6-sgRNA-scaffold expression cassette. Duplicated T-rich regions, poly-purine tract (PPT), and LTR sequences are highlighted in red. **b,** representative immunoblot showing Nef and GAPDH expression levels in HEK293T cells transfected with the HIV-1 CH077 constructs. **c,** Infectious virus yield after transfection of HEK293T cells with the parental HIV-1 CH077 construct (back), the original (darker pink) or optimized (pink) containing the U6-sgRNA-scaffold cassette or the sgRNA library. Bars represent the mean of four independent experiments ±SEM. Unpaired T-test with Welch´s correction, two-tailed. *p<0.05, ** p<0.001, ***p<0.0001. **d,** Read counts in virus stock correlated with read counts in the proviral DNA of the HIV-1 TV-CH077-CRF-sgRNA library. **e**, Replication kinetics of the HIV-1 TV-CH077-CRF-sgRNA library in in CEM-M7 Cas9 cells. Infected cells were quantified by flow cytometry (left) and infectious virus in the supernatant by TZM-bl infection assay (right) in absence (black) or presence (pink) of IFN-β. Dots represent the mean of two (left panel) or three (right panel) measurements ± SEM. **f,** Representative gating strategy of the data in (e).

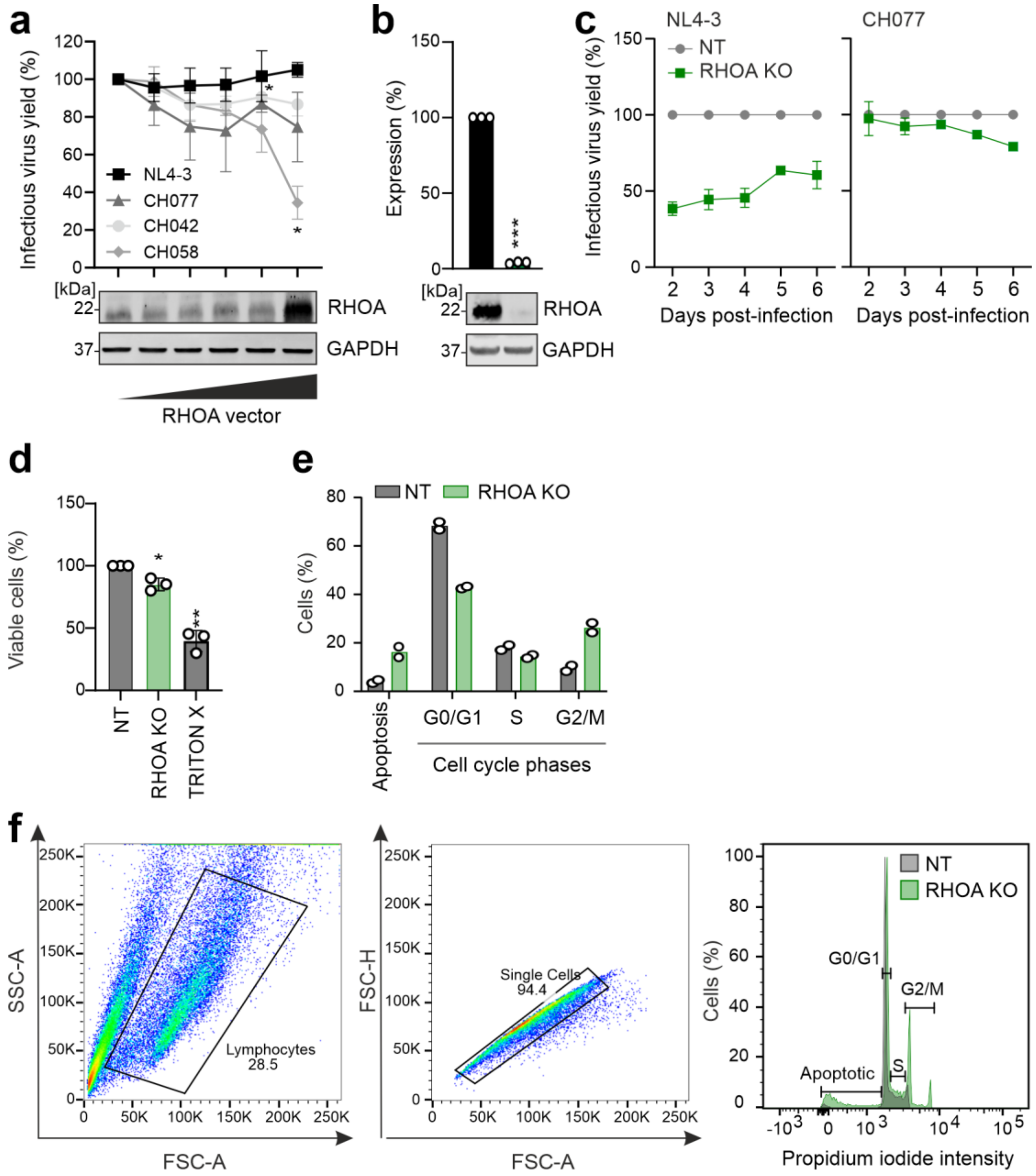

Supplementary Figure 7. RHOA KO impairs HIV-1 replication and inhibits cell cycling. **a,** Effect of RHOA overexpression on the indicated HIV-1 proviral constructs. Values represent mean of three independent experiments ±SEM. **b,** RHOA expression upon KO in primary CD4$^+$ T cells. Bars represent the mean of three donors ±SD. **c,** Impact of RHOA KO (green) on infectious virus production by CD4$^+$ T cells infected with the indicated WT HIV-1 strains. NT sgRNA set to 100% (grey). Values represent the mean of two experiments ±SEM. **d,** Percentages of viable cells as measured by MTT assay of the experiment in panel (b). NT (grey), RHOA KO (green), TritonX (dark grey). **e,** Percentages of primary CD4$^+$ T cells electroporated with either sgRNA targeting RHOA (green) or the NT control (grey) and stained with Propidium iodide (PI) at indicated cell cycle phases. Bars represent the mean of two donors. **f,** Representative gating strategy of the data in (e). b, d Unpaired T-test Welch´s correction, two-tailed. *p<0.05, ***p<0.0001.

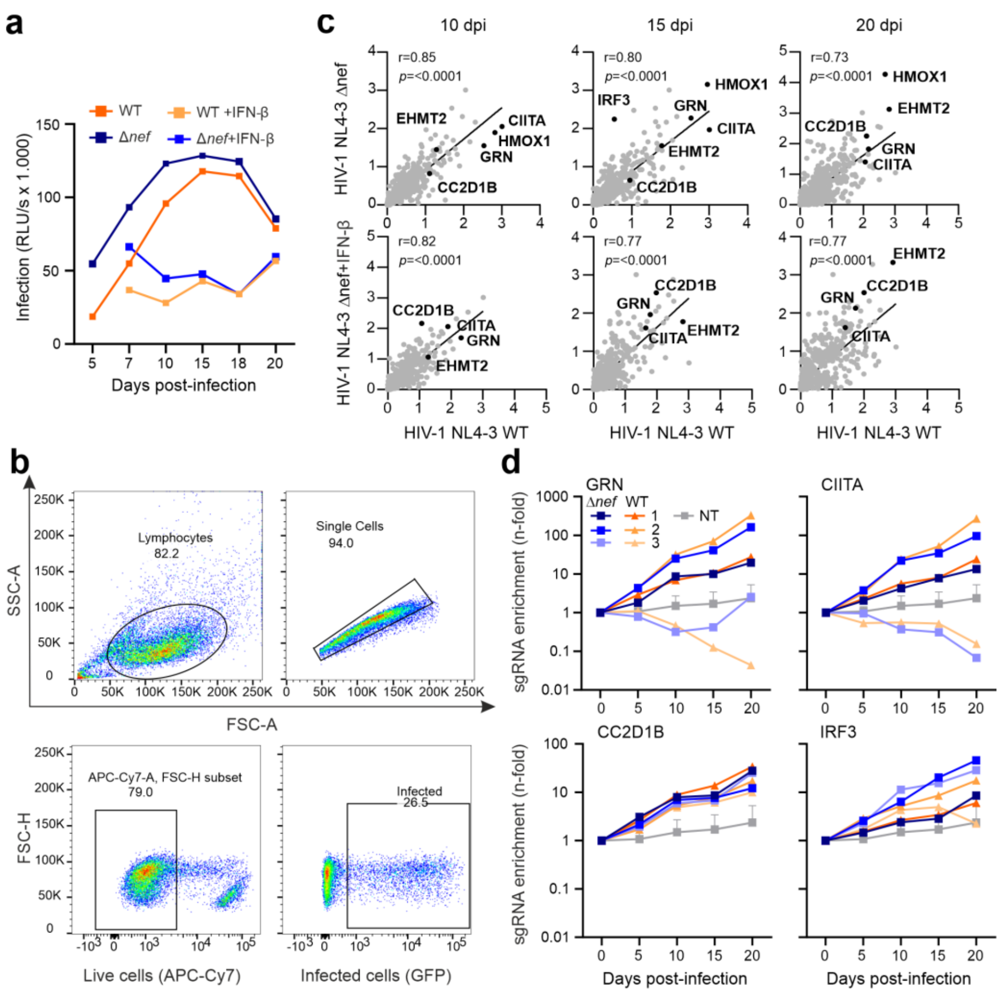

Supplementary Figure 8. Spread and enrichment of HIV-1 CRF-sgRNA Δ*nef* constructs in CEM-M7 Cas9 cells. a, Infectious virus yields in CEM-M7 Cas9 cells infected with the TV-NL4-3-CRF-sgRNA WT (orange) or Δ*nef* library (blue). Cells were infected and the virus passaged as indicated in Figure 1a. **b,** Representative gating strategy of the data in (a). **c,** Correlation between the enrichment of genes at the 10, 15 and 20 day time-points between the WT and Δ*nef* kinetics in absence (upper) and presence (lower) of IFN-β treatment. Dots of selected genes are shown in black. **d,** Read counts relative to input virus from the MAGeCK analysis showing the enrichment of sgRNAs targeting *GRN*, *CIITA*, *CC2D1B* and *IRF3* in Δ*nef* (blue) and WT (orange) kinetics. NT control (grey) represents the mean of 11 NT gRNAs±SEM.

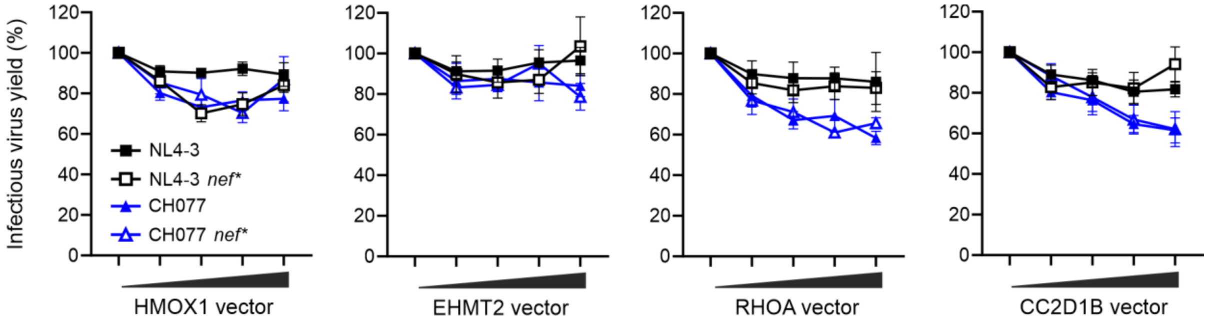

Supplementary Figure 9. HMOX1, EHMT2, RHOA and CC2D1B are not antagonized by Nef. HEK293T cells were cotransfected with increasing amounts of the indicated expression constructs and WT or *nef*-defective NL4-3 (black) or CH077 (blue) proviral constructs. Points represents the mean of 6 independent experiments ±SEM.

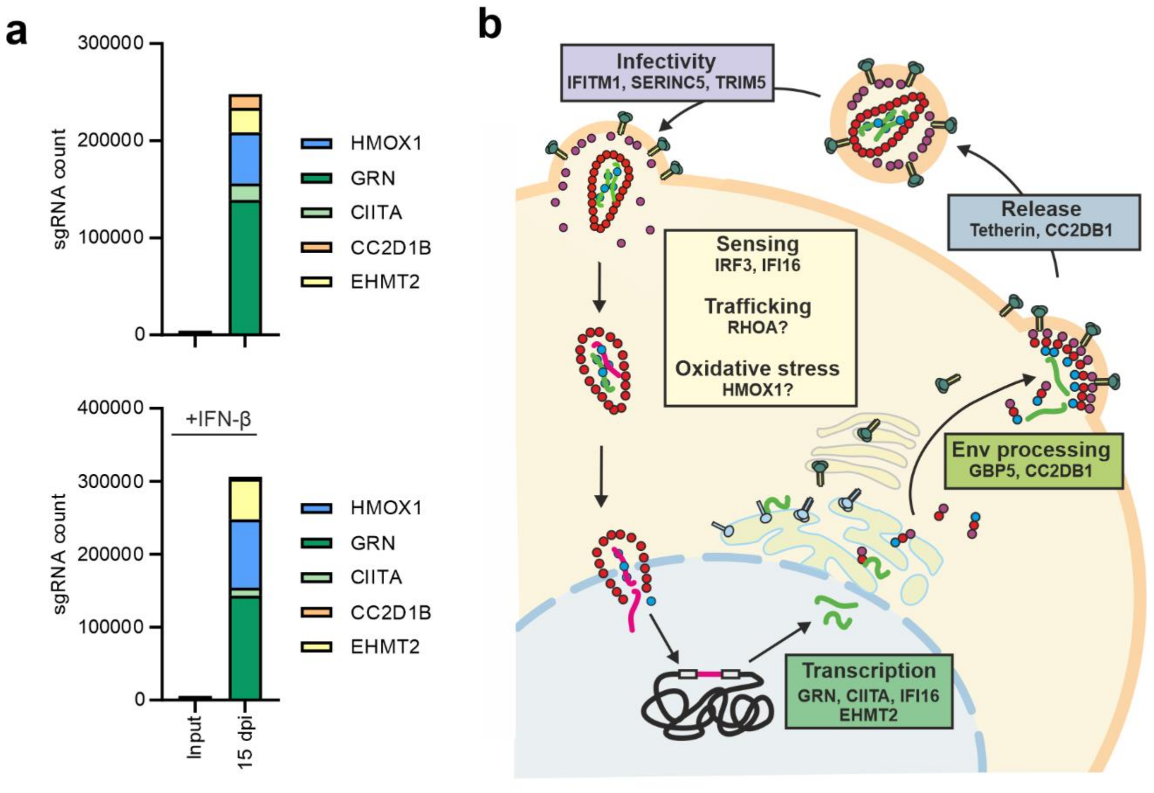

Supplementary Figure 10. Overview on HIV-1-driven selection of sgRNAs and their cellular targets. a, Stacking plot of the enrichment of selected sgRNA counts from input to 15 days post infection of CEM-M7 Cas9 cells, untreated (upper panel) or treated with IFN-β (lower panel). Shown are HMOX1 (light blue), GRN (green), CIITA (light green), CC2D1B (orange) and EHMT2 (yellow). **b,** Schematic overview of the HIV-1 replication cycles and cellular factors expressed by genes target by sgRNA that became strongly enriched by the TV-driven screening approach. The exact inhibitory mechanisms of some of the highlighted cellular factors remain to be determined.
