# Supplementary material for: Replication competent HIV-guided CRISPR screen identifies antiviral factors including targets of the accessory protein Nef
Source: Nat Commun. 2024 May 7;15:3813. doi: 10.1038/s41467-024-48228-x (PMC11076291; doi:10.1038/s41467-024-48228-x)
Supplement: Supplementary file 3 — Description of Additional Supplementary Files [file 41467_2024_48228_MOESM3_ESM.docx]

**Description of Additional Supplementary Files**

**File Name: Supplementary Data 1**

**Description:** **List of gRNAs**

**File Name: Supplementary Data 2**

**Description: Sequencing primers**

**File Name: Supplementary Data 3**

**Description: Raw P values**
