# Supplementary material for: Replication competent HIV-guided CRISPR screen identifies antiviral factors including targets of the accessory protein Nef
Source: Nat Commun. 2024 May 7;15:3813. doi: 10.1038/s41467-024-48228-x (PMC11076291; doi:10.1038/s41467-024-48228-x)
Supplement: Supplementary file 7 — Reporting Summary [file 41467_2024_48228_MOESM7_ESM.pdf]

nature portfolio

Corresponding author(s): Frank Kirchhoff
Konstantin M.J. Sparrer

Last updated by author(s): 10.04.2024

Reporting Summary

Nature Portfolio wishes to improve the reproducibility of the work that we publish. This form provides structure for consistency and transparency in reporting. For further information on Nature Portfolio policies, see our Editorial Policies and the Editorial Policy Checklist.

Statistics

For all statistical analyses, confirm that the following items are present in the figure legend, table legend, main text, or Methods section.

n/a	Confirmed	
☐	☒	The exact sample size (*n*) for each experimental group/condition, given as a discrete number and unit of measurement
☐	☒	A statement on whether measurements were taken from distinct samples or whether the same sample was measured repeatedly
☐	☒	The statistical test(s) used AND whether they are one- or two-sided<br>*Only common tests should be described solely by name; describe more complex techniques in the Methods section.*
☒	☐	A description of all covariates tested
☒	☐	A description of any assumptions or corrections, such as tests of normality and adjustment for multiple comparisons
☐	☒	A full description of the statistical parameters including central tendency (e.g. means) or other basic estimates (e.g. regression coefficient) AND variation (e.g. standard deviation) or associated estimates of uncertainty (e.g. confidence intervals)
☐	☒	For null hypothesis testing, the test statistic (e.g. *F*, *t*, *r*) with confidence intervals, effect sizes, degrees of freedom and *P* value noted<br>*Give P values as exact values whenever suitable.*
☒	☐	For Bayesian analysis, information on the choice of priors and Markov chain Monte Carlo settings
☒	☐	For hierarchical and complex designs, identification of the appropriate level for tests and full reporting of outcomes
☒	☐	Estimates of effect sizes (e.g. Cohen's *d*, Pearson's *r*), indicating how they were calculated

Our web collection on statistics for biologists contains articles on many of the points above.

Software and code

Policy information about availability of computer code

Data collection	BD FACSDiva™ Version 8.0, LI-COR Image Studio Version 5.2, StepOnePlus Real-Time PCR System software
Data analysis	GraphPad Prism Version 10, Corel DRAW 23.1, LI-COR Image Studio Lite Version 5.0.21, FlowJo 10, RStudio 2022.12.0, Galaxy version 23.0, SeqPrep 0.2.2, MAGeCK 0.5.9.2.4

For manuscripts utilizing custom algorithms or software that are central to the research but not yet described in published literature, software must be made available to editors and reviewers. We strongly encourage code deposition in a community repository (e.g. GitHub). See the Nature Portfolio guidelines for submitting code & software for further information.

Data

Policy information about availability of data

All manuscripts must include a data availability statement. This statement should provide the following information, where applicable:

- Accession codes, unique identifiers, or web links for publicly available datasets
- A description of any restrictions on data availability
- For clinical datasets or third party data, please ensure that the statement adheres to our policy

A data availability statement is included, Raw data is provided in the source file or on GEO (see data availability statement).

Research involving human participants, their data, or biological material

Policy information about studies with human participants or human data. See also policy information about sex, gender (identity/presentation), and sexual orientation and race, ethnicity and racism.

Reporting on sex and gender	N.A.
Reporting on race, ethnicity, or other socially relevant groupings	N.A.
Population characteristics	Healthy humans that donate blood, further information and approval is provided in the manuscript.
Recruitment	Healthy human donors donating blood under 30 years old.
Ethics oversight	Ethikkommission der Universität Ulm

Note that full information on the approval of the study protocol must also be provided in the manuscript.

Field-specific reporting

Please select the one below that is the best fit for your research. If you are not sure, read the appropriate sections before making your selection.

- [x] Life sciences
- [ ] Behavioural & social sciences
- [ ] Ecological, evolutionary & environmental sciences

For a reference copy of the document with all sections, see nature.com/documents/nr-reporting-summary-flat.pdf

Life sciences study design

All studies must disclose on these points even when the disclosure is negative.

Sample size	No sample size calculations were performed. Based on previous experiences, results were confirmed in at least two - three independent experiments.
Data exclusions	no data was excluded
Replication	The number of independent replicates to similar results is indicated in the respective figure legends or the Statistics and Reproducibility section to keep the main figure legends concise. All technically sound attempts at replication yielded similar results.
Randomization	Randomization was not applicable for this study, as no human trials or cohort studies were performed or samples assigned to experimental groups.
Blinding	Blinding was not applicable, data was collected using automated procedures.

Reporting for specific materials, systems and methods

We require information from authors about some types of materials, experimental systems and methods used in many studies. Here, indicate whether each material, system or method listed is relevant to your study. If you are not sure if a list item applies to your research, read the appropriate section before selecting a response.

Materials & experimental systems

n/a	Involved in the study	
[ ]	[x]	Antibodies
[ ]	[x]	Eukaryotic cell lines
[x]	[ ]	Palaeontology and archaeology
[x]	[ ]	Animals and other organisms
[x]	[ ]	Clinical data
[x]	[ ]	Dual use research of concern
[x]	[ ]	Plants

Methods

n/a	Involved in the study	
[x]	[ ]	ChIP-seq
[ ]	[x]	Flow cytometry
[x]	[ ]	MRI-based neuroimaging

Antibodies

Antibodies used	IRDye® 680RD Goat anti-Rabbit IgG (H + L) LI-COR Cat# 926-68071 (1:20000);<br>IRDye® 800CW Goat anti-Mouse IgG (H + L) LI-COR Cat# 926-32210 (1:20000);<br>IRDye® 800CW Goat anti-Rabbit IgG (H + L) LI-COR Cat# 926-32211(1:20000);

IRDye 800RD Goat anti-Rat IgG (H + L) LI-COR Cat# 925-32219 (1:20000);
IRDye 680RD Goat anti-Rat IgG (H + L) LI-COR Cat# 926-68071 (1:20000);
Recombinant Anti-Granulin Abcam Cat# ab208777 (1:200);
Monoclonal Mouse Anti CIITA Santa Cruz Cat#sc-13556 (1:200);
Monoclonal Anti CEACAM3 Abcam Cat# ab196606 (1:200);
Monoclonal Anti CC2D1B Proteintech Cat# 20774-1-AP (1:200);
Monoclonal Anti HMOX1 Sigma Cat# # MA1-112 (1:200);
Monoclonal Rabbit Anti ISG15 Santa Cruz Cat# sc-166755 (1:1000);
Monoclonal Rabbit Anti EHMT2 Cell signaling Cat#3306 (1:200);
Monoclonal Anti IFI16 Santa Cruz Cat# sc-8023 (1:150);
Monoclonal Anti-Human Immunodeficiency Virus Type 1 (HIV-1) Nef Protein NIH AIDS reagents program Cat# ARP1539 (1:1000);
Monoclonal Mouse Anti RHOA Abcam Cat#ab54835 (1:200);
HIV-1 p24 Abcam Cat# ab9071 (1:1000);
HIV-1 Env NIH AIDS reagents program Cat# ARP-12559 (1:1000);
Polyclonal Rabbit Anti BST-2 Proteintech Cat# 13560-1-AP (1:1000);
Goat monoclonal anti-GBP5, Santa Cruz Cat# sc-1603539 (1:150);
Polyclonal Anti-GBP5 Proteintech Cat# 13220-1-AP (1:1000);
Mouse anti-Cas9(s.pyogenes) Cell signaling Cat#14697 (1:1000);
Rat monoclonal anti-GAPDH Biolegend Cat# 607902 (1:1000);
Anti-HIV-1 p24 core antigen-RD1, KC57 Beckman Coulter Cat# :6604667 (1:100);
PerCP/Cyanine5.5 anti-human CD4 Antibody Biolegend Cat#317428 (1:50);
Mouse anti-Cas9 (7A9-3A3) Alexa Fluor® 647 Conjugate Cell signaling Cat#48796 (1:100);
Goat anti-rabbit PE Abcam Cat#ab97070 (1:100);
Mouse (MOPC-21) anti-IgG1 Isotype Control Alexa Fluor® 647 Conjugate Cell signaling Cat#4843 (Concentration dependent);
eBioscience™ Fixable Viability Dye eFluor™ 780 ThermoFisher Cat# 65-0865-14 (1:1000)
FITC mouse anti-Human CD25 BD Pharmigen Cat# 555431 (1:5)
PE-Cy5 mouse anti-Human HLA-DR BD Pharmigen Cat# 555813 (1:5)
Alexa Fluor 647 mouse anti-Human CD66d/e Biolegend Cat#392806 (1:100)
Alexa Fluor 647 mouse IgG1 isotype control Santa Cruz Cat# sc-24636 (1:40)

Validation

Abcam:
Antibody Validation for Western blot
Antibodies are validated in western blot using lysates from cells or tissues that we have identified to express the protein of interest. Once we have determined the right lysates to use, western blots are run and the band size is checked for the expected molecular weight. We will always run several controls in the same western blot experiment, including positive lysate and negative lysate.
When possible, we also include knock-out (KO) cell lines as a true negative control for our western blots. We are always increasing the number of KO-validated antibodies we provide. In addition, we run old stock alongside our new stock. If we know the old stock works well, this also acts as a suitable positive control.
If the western blot result gives a clear clean band and we are happy with the result from the control lanes, these antibodies will be passed and added to the catalog.
Cell Signalling:
Antibody Validation for Immunofluorescence:
Cell lines or tissues with known target expression levels are used to verify specificity.
Appropriate cell lines and tissues are used to verify subcellular localization.
Antibody performance is assessed on appropriate tissues.
Cells are subjected to phosphatase treatment to verify phospho-specificity. Target specificity is also verified with the use of known knockout or null cell lines.
Cells are subjected to siRNA treatment or over-expression of the target protein to verify target specificity.
Activation state specification, target expression, and translocation are examined using ligands or inhibitors to modulate pathway activity.
Requirement of threshold signal-to-noise ratio in antibody:isotype comparison and minimum fold-induction for phospho-specific antibodies ensures the greatest possible sensitivity.
Fixation and permeabilization conditions are optimized; alternative protocols are recommended if necessary.
Stringent testing ensures lot-to-lot consistency.
Antibody Validation for Western Blotting:
Examination of several cell lines and/or tissues of known expression levels allows accurate determination of species cross-reactivity and verifies specificity.
Treatment of cell lines with growth factors, chemical activators or inhibitors, which induce or inhibit target expression, verifies specificity. Phosphatase treatment confirms phospho-specificity.
The use of siRNA transfection or knockout cell lines verifies target specificity.
Side-by-side comparison of lots to ensures lot-to-lot consistency.
Optimal dilutions and buffers are predetermined, positive and negative cell extracts are specified, and detailed protocols are already optimized, saving valuable time and reagents.
Sigma Aldrich:
The monoclonal antibody detects only the target protein band(s) on a Western blot from an E. coli, plant or mammalian crude cell lysate. The monoclonal antibody detects as little as 2 ng of target protein by dot blot. The Western blot is tested down to 10 ng, but may detect lower using the procedure detailed below.
Bio-Techne/ Novus Biologicals:
For western blot, specificity and sensitivity can be done by choosing known positive and negative models. In the case of phosphorylated targets, kinase activators and inhibitors can be utilized. In some cases, low expression levels of a specific protein

combined with closely related proteins within the same family make it very challenging to test the antibody panels. This may require the utilization of transfectant models to further investigate the reactivity of a chosen clone. Western blot data that show specificity with the expected molecular weight and expression profile are a very useful tool to support other applications such as IHC and flow cytometry. However, since some applications have antibodies that recognize folded epitopes, many flow cytometry or assay reagents are not compatible with western blot. Therefore, western blot results cannot be used as supportive evidence for these applications. This is where the careful choice of positive and negative cell models is crucial to the validation of the reagent. For IHC, that may require testing across various tissue or cancer types, to exclude off-target reactivity. Upon completion of testing across all required applications, the data is reviewed by a team of scientists and the best performing clones are made available commercially. R&D Systems' antibodies are tested far beyond simple positive validation via western blot or ICC, as is common for many commercially available antibodies. Prior to relese, the production and bottled lots undergo a stringent quality control test, running the optimized conditions for the various applications under ISO9001 quality controlled document management.
Bio Legend:
As knocking out the target protein is one of the most trusted antibody validation processes, we are starting to validate our Cell Biology portfolio antibodies by KO (knockout) and KD (knockdown) systems. To confirm antibody specificity, Western blot data using BioLegend's in-house generated CRISPR/Cas9 and siRNA, as well as CRISPR/Cas9 KO cell lysates from a collaboration with EdiGene (a genome editing company) will be made readily available to researchers
Proteintech:
Every antibody is extensively validated by an in-house team of scientists using unmodified samples and endogenous levels of proteins. With the increasing need for antibodies with high specificity and reproducibility, Proteintech introduced siRNA knockdown validation, one of the most trusted and accepted forms of antibody validation. Look out for the siRNA Knockdown symbol across the Proteintech catalog.
Origene:
Specificity validation is performed via predicted band detected in Western blot analysis, idependent antibody strategies, 10k protein chip and w estern blot using Knockout cell lysates.
Bio-Rad:
Validation methods, including knockout (KO) validation, knockdown (siRNA) validation and immunoprecipitation followed by mass spectrometry (IP-MS) are used.

Eukaryotic cell lines

Policy information about cell lines and Sex and Gender in Research

Cell line source(s)	Human HEK293T cells ATCC Cat# CRL-3216<br>TZM-bl NIH Cat#8129<br>CEM-M7 Cas9 This paper<br>SupT1-CCR5 high-Cas9 This paper
Authentication	The cell lines were authenticated by ATCC, NIH or their lab of origin and not validated further in our laboratory.
Mycoplasma contamination	Cells were tested routinely to be free of mycoplasma using a PCR based test.
Commonly misidentified lines (See ICLAC register)	No commonly misidentified cell lines were used.

Plants

Seed stocks	N.A.
Novel plant genotypes	N.A.
Authentication	N.A.

Flow Cytometry

Plots

Confirm that:

- [ ] The axis labels state the marker and fluorochrome used (e.g. CD4-FITC).
- [ ] The axis scales are clearly visible. Include numbers along axes only for bottom left plot of group (a 'group' is an analysis of identical markers).
- [ ] All plots are contour plots with outliers or pseudocolor plots.
- [x] A numerical value for number of cells or percentage (with statistics) is provided.

Methodology

Sample preparation	To monitor infection during the replication kinetic, flow cytometry was used to quantify the infected cells. For CEM-M7 Cas9 kinetics, ~400,000 cells were harvested, washed once with PBS and stained for 15min at RT in the dark with eBioscience Fixable viability dye 780 (ThermoFisher Scientific) diluted 1:1000 in PBS. Cells were washed twice with PBS and fixed in 2% PFA for 30 min at 4°C. For SupT1 CCR5 high Cas9 kinetic and to monitor KO efficiencies in cells infected with HIV-1 either carrying the NT or BST2 or GBP5 gRNA, ~400,000 cells were harvested, washed once with PBS and stained for 30 min at RT in the dark with anti-CD4 antibody (Biolegend) diluted 1:50 in PBS and eBioscience Fixable viability dye 780 (ThermoFisher Scientific) diluted 1:1000 in PBS. Afterwards cells were washed twice with PBS and permeabilized 20 minutes with 200µl BD Cytofix/Cytoperm at RT. Cells were washed twice with 200µl BD 1X Perm/Wash and stained 1h at 4°C with anti-HIV-1 p24 (Beckman Coulter, KC57) or anti-BST2 or anti-GBP5 antibodies diluted 1:100 in 1X Perm/Wash. After washing twice with 1X Perm/Wash, wells were either stained with secondary antibody antibody goat anti rabbit PE (1:100 in 1X Perm/Wash) or fixed in 2% PFA for 30 min at 4°C.
Instrument	FACS Calibur or FACS Canto; BD
Software	FlowJo 10
Cell population abundance	Single living cells (90%)
Gating strategy	For the kinetics, the gating strategy was always the following: all cells SSC-A / FSC-A, single cells FSC-A / FSC-H, alive cells (APC-CY7+), Infected cells (GFP+ or p24 (PE) positive/CD4(PerCP-Cy5.5) negative cells. Raw fluorescence-activated cell sorting (FACS) data were analysed using FlowJo 10

- [x] Tick this box to confirm that a figure exemplifying the gating strategy is provided in the Supplementary Information.
